# Virulence of *Acinetobacter baumannii* Exhibiting Phenotypic Heterogeneous Growth against Meropenem in a Murine Thigh Infection Model

**DOI:** 10.3390/antibiotics2010073

**Published:** 2013-03-11

**Authors:** Evangelia Neou, George Michail, Athanassios Tsakris, Spyros Pournaras

**Affiliations:** 1 Department of Microbiology, Medical School, University of Thessaly, Larissa 41110, Greece; E-Mails: lilianneou@yahoo.com (E.N.); michailgeorge@hotmail.com (G.M.); 2 Department of Microbiology, Medical School, University of Athens, Athens 11527, Greece; E-Mail: atsakris@med.uoa.gr

**Keywords:** heteroresistance, persisters, virulence, experimental infections

## Abstract

*Acinetobacter baumannii* may exhibit phenotypic heterogeneous growth under exposure to antibiotics. We investigated the *in vitro* characteristics of *A. baumannii* isolates grown heterogeneously in the presence of meropenem and their virulence evaluated in experimental infections treated with meropenem. Five clinical *A. baumannii* isolates and the respective heterogeneously grown subpopulations were tested by agar dilution minimum inhibitory concentration (MIC) testing, pulsed field gel electrophoresis (PFGE), population analysis using meropenem and growth curves. The virulence of isolates and the therapeutic efficacy of three meropenem dosing schemes was evaluated in a neutropenic murine thigh infection model. The clinical isolates were meropenem-susceptible (MICs 1 to 4 mg/liter) and exhibited three distinct PFGE patterns. In all clinical isolates, population analysis yielded heterogeneously grown colonies. After seven subcultures in antibiotic-free media, resistant MIC levels were retained in two isolates (heteroresistant), while three isolates were reversed to susceptible MICs (persisters). Clinical isolates and heterogeneous subpopulations had similar growth rates. The heterogeneously grown *A. baumannii* subpopulations had reduced virulence, killing considerably fewer animals than the respective clinical isolates without treatment. The meropenem treatment outcome was similar in infections caused by the clinical and the heterogeneous isolates, irrespective to their MICs. *In vitro* meropenem exposure induces phenotypic heterogeneous growth in *A. baumannii*. Compared with the parental clinical isolates, the heterogeneously grown subpopulations exhibited lower virulence, killing fewer mice and responding equally to meropenem treatment, despite their higher MICs.

## 1. Introduction

*Acinetobacter baumannii* has been an important nosocomial pathogen for the past 30 years, frequently implicated in ventilator-associated pneumonia, bloodstream infections and urinary tract infections [1]. Ubiquity and propensity to develop antibiotic resistance make *A. baumannii* a common, yet difficult-to-treat, hospital pathogen [2], which often needs the use of carbapenems as a treatment of last resort. 

During the last few years, reduced susceptibility or resistance to carbapenems is increasingly observed among *A. baumannii* clinical isolates [3,4]. A further worrisome observation is heteroresistance of *A. baumannii* to antibiotics, such as colistin or carbapenems [5,6,7], which may have implications for the treatment of *A. baumannii* infections. We have previously demonstrated that meropenem pressure on *A. baumannii* can produce subpopulations with heterogeneous expression of resistance, reflected by colonies grown within the zone of inhibition around meropenem disks or Etest strips [7,8]. These colonies may represent truly resistant subpopulations with stable changes in their genome associated with antimicrobial resistance (heteroresistance), or they may be related to subpopulations able to persist in a latent state in the presence of antimicrobials (bacterial persistence) [9]. Inherent heterogeneity of bacterial populations may contribute to their adaptation to fluctuating environments and to the persistence of bacterial infections [10]. 

So far, there are scarce reports of experimental infections caused by *A. baumannii* and treated with carbapenems, such as meropenem [11]. Also, to the best of our knowledge, there is no data on the therapeutic activity of meropenem against infections caused by *A. baumannii* with phenotypic heterogeneous growth against carbapenems. We present herein the characteristics of *A. baumannii* clinical isolates with phenotypic heterogeneous growth against meropenem, as well as their infectivity and the meropenem therapeutic efficacy evaluated in neutropenic murine thigh infections. 

## 2. Results and Discussion

### 2.1. Phenotypic Assays

The characteristics of the study isolates are shown in Table 1. All clinical isolates were initially classified as meropenem-susceptible by the Vitek2 system and exhibited susceptible agar dilution MICs for meropenem that ranged from 1 to 4 mg/L. However, population analysis assays yielded colonies that grew in the presence of meropenem at 8 to 32 mg/L for all clinical isolates. All isolates were susceptible to colistin and exhibited various susceptibility profiles to other antimicrobials. 

**Table 1:** Characteristics of the study isolates.

Isolate	Ward	Specimen	^a ^Susceptibility status to antimicrobials	PFGE	Agar dilution MEM MIC (mg/L)	Highest MEM concentration of growth in population analyses (mg/L)	^b ^Agar dilution MIC (mg/L) of MEM grown subpopulations
Ab1	ORL	Blood	SAM, CIP, COL, MEM	I	4	16	32
Ab2	Neurosurgery	Blood	GEN, TOB, CIP, SAM, MEM, COL	II	1	8	1
Ab3	Pathology	Urine	GEN, TOB, SAM, MEM, COL	I	2	8	1
Ab4	Pulmonary	Sputum	GEN, SAM, MEM, COL	I	4	16	2
Ab5	Pathology	CSF	GEN, TOB, SAM, MEM, COL	III	2	32	> 32

^a^ CIP, ciprofloxacin; COL, colistin; GEN, gentamicin; MEM, meropenem; MIC, minimum inhibitory concentration; PFGE, pulsed field gel electrophoresis; SAM, ampicillin/sulbactam; TOB, tobramycin; ^b^ These MICs were estimated after one week of daily subcultures in antibiotic-free medium.

The growth rates of the clinical isolates did not differ considerably from those of the heterogeneously grown subpopulations (data not shown).

After seven daily subcultures in antibiotic-free medium, the colonies of two clinical isolates (Ab1 and Ab5) that grew in the highest meropenem concentration, exhibited stable meropenem resistance (heteroresistance). In the remaining three clinical isolates (Ab2, Ab3 and Ab4), heterogeneously grown colonies were reversed to low meropenem MICs, which were similar to those of the respective clinical isolates (persistence). 

### 2.2. PFGE and Polymerase Chain Reaction (PCR) Assays

PFGE analysis discriminated three distinct genotypes among the *A. baumannii* clinical isolates, with the patterns of the heterogeneously grown subpopulations to be indistinguishable from those of the respective clinical isolates. PCR was positive only for the intrinsic *bla*_OXA-51-_like carbapenemase, while it was negative for IS*Aba*1 elements upstream of the *bla*_OXA-51_-like carbapenemase gene and also for genes encoding acquired carbapenemases.

### 2.3. Thigh Infection Model

The results of the experimental infections are presented in Table 2. 

*Untreated mice.* The experimental infections were initially performed without treatment, to estimate the virulence potential of the study isolates and the respective heterogeneous subpopulations. All 15 mice that were infected by the five clinical isolates and were not given treatment died within 24 h. The six untreated mice infected by two meropenem-persisting subpopulations (Ab3 and Ab4) also died within 24 h, while the nine mice infected by one persister and two heteroresistant subpopulations (Ab1h, Ab2h and Ab5h) survived 24 h. These results indicate a relatively higher virulence of the clinical isolates compared with the heterogeneously grown subpopulations.

**Table 2:** Results of the experimental infections.

Strain		Treatment regimen	Mean bacterial lung concentration (log CFU/g thigh)	Mortality (n)
Ab1	Clinical isolate	Untreated control	10.468	3/3
		Meropenem 20 mg /kg/8 h	10.250	1/3
		Meropenem 100 mg/kg/12 h	7.992	1/3
		Meropenem 400 mg/kg/8 h	8.480	0/3
Ab1h	Heterogeneous subpopulation	Untreated control	10.079	0/3
		Meropenem 20 mg /kg/8 h	10.010	0/3
		Meropenem 100 mg/kg/12 h	7.599	0/3
		Meropenem 400 mg/kg/8 h	6.135	0/3
Ab2	Clinical isolate	Untreated control	10.568	3/3
		Meropenem 20 mg/kg/8 h	10.450	2/3
		Meropenem 100 mg/kg/12 h	10.508	0/3
		Meropenem 400 mg/kg/8 h	7.393	0/3
Ab2h	Heterogeneous subpopulation	Untreated control	10.560	0/3
		Meropenem 20 mg /kg/8 h	10.450	0/3
		Meropenem 100 mg/kg/12 h	10.508	0/3
		Meropenem 400 mg/kg/8 h	7.393	0/3
Ab3	Clinical isolate	Untreated control	10.370	3/3
		Meropenem 20 mg/kg/8 h	10.250	2/3
		Meropenem 100 mg/kg/12 h	10.032	0/3
		Meropenem 400 mg/kg/8 h	8.100	0/3
Ab3h	Heterogeneous subpopulation	Untreated control	10.365	3/3
		Meropenem 20 mg /kg/8 h	10.125	0/3
		Meropenem 100 mg/kg/12 h	10.032	1/3
		Meropenem 400 mg/kg/8 h	8.124	0/3
Ab4	Clinical isolate	Untreated control	10.280	3/3
		Meropenem 20 mg/kg/8 h	10.145	1/3
		Meropenem 100 mg/kg/12 h	10.055	0/3
		Meropenem 400 mg/kg/8 h	7.950	0/3
Ab4h	Heterogeneous subpopulation	Untreated control	10.412	3/3
		Meropenem 20 mg /kg/8 h	10.275	1/3
		Meropenem 100 mg/kg/12 h	10.032	0/3
		Meropenem 400 mg/kg/8 h	8.116	0/3
Ab5	Clinical isolate	Untreated control	10.350	3/3
		Meropenem 20 mg/kg/8 h	10.150	2/3
		Meropenem 100mg/kg/12 h	10.035	1/3
		Meropenem 400 mg/kg/8 h	8.066	0/3
Ab5h	Heterogeneous subpopulation	Untreated control	10.128	0/3
		Meropenem 20 mg /kg/8 h	10.078	0/3
		Meropenem 100 mg/kg/12 h	10.032	0/3
		Meropenem 400 mg/kg/8 h	8.299	0/3
*E. coli* ATCC 25922		Untreated control	9.077	0/3
		Meropenem 20 mg/kg/8 h	8.015	0/3
		Meropenem 100 mg/kg/12 h	7.979	0/3
		Meropenem 400 mg/kg/8 h	2.015	0/3

*Mice treated with 20 mg/kg meropenem.* Experimental data relative to carbapenem dosing schemes for the treatment of infections due to *A. baumannii* were very limited, and there was only one study that applied 20 mg/kg meropenem [11]. Under this meropenem regimen, 8/15 mice infected with clinical isolates died within 24 h, in contrast with only 1/15 mice dying when infected with heterogeneous subpopulations, also indicating a relatively higher virulence of the clinical isolates. No significant decrease in colonies grown was observed for all 30 mice that received 20 mg/kg treatment compared with untreated ones (*p* > 0.05), suggesting a poor efficacy of this dosing scheme.

*Mice treated with 100 mg/kg meropenem.* Since the above treatment regimen of 20 mg/kg exhibited very poor outcomes in the initial experiments, we applied a higher dose of 100 mg/kg that was used in murine infections caused by another Gram-negative non-fermenting species, *Pseudomonas aeruginosa* [12]. The outcome of 100 mg/kg meropenem treatment was similar for the clinical isolates and the heterogeneously grown subpopulations (13/15 mice infected by clinical isolates and 14/15 mice infected by heterogeneous subpopulations survived 24 h), despite the higher MICs of the latter populations. No significant decrease in colonies grown was observed for all 30 mice given 100 mg/kg treatment compared with untreated ones (*p* > 0.05), also suggesting the poor efficacy of this treatment.

*Mice treated with 400 mg/kg meropenem*. Another dosing scheme previously used also against pseudomonal murine infections [13] was further tested. All 30 mice infected by the clinical isolates and by the heterogeneously grown subpopulations survived 24 h under 400 mg/kg meropenem treatment. A significant decrease (*p* < 0.05) in colony counts was observed for 12/15 mice infected by four clinical isolates and for all 15 mice infected by heterogeneous subpopulations, compared with untreated mice, suggesting therapeutic efficacy of this dosing scheme. For the three mice infected by the clinical isolate Ab1, no significant decrease in colonies grown was observed, despite treatment, compared with untreated ones (*p* > 0.05), although Ab1 was meropenem-susceptible, indicating its higher infectivity compared with the respective heterogeneous subpopulation that responded more favorably even being meropenem-resistant.

In contrast with the *A. baumannii* isolates, all 12 mice infected by the *Escherichia coli* ATCC 25922 strain survived 24h with 20, 100 and 400 mg/kg meropenem or without treatment. A significant decrease (*p* < 0.05) in colony counts was observed only for the highest used dosing scheme. During the last few years, *A. baumannii* isolates that exhibit carbapenem resistance are increasingly isolated and pose substantial therapeutic problems in many regions worldwide [14]. Furthermore, the ability of *A. baumannii* cells to survive under considerably higher carbapenem concentrations (phenotypic heterogeneous growth) may have implications for the treatment of multiresistant *A. baumannii* infections [15] and poses concerns. The observations of a previous study suggested that *A. baumannii* isolates that are apparently meropenem-susceptible by standard susceptibility testing may contain a certain amount of phenotypically meropenem-resistant subpopulations [7]. These subpopulations may have stably elevated meropenem MICs due to permanent genomic changes and are considered heteroresistant or may simply persist in a latent state in the presence of antimicrobials and exhibit susceptible MICs when they grow without meropenem exposure (persisters) [10]. 

Drug resistance in bacteria can be associated with a biological fitness cost [16]. It has been proposed that the magnitude of this cost is the primary factor that influences the rate of resistance development, the stability of the resistance and the rate at which the resistance might decrease if antibiotic use were reduced. However, mutants with no measurable cost have also been observed. It has been proposed that environmental conditions affect fitness cost and the cost of drug resistance can be reduced by regulation of the resistance mechanism or cost compensation [17].

In the present study, heteroresistant phenotype was observed in two isolates, which retained meropenem MICs of 32 mg/L or higher, while three isolates exhibited a persister phenotype, with MICs to return to the susceptible range after seven daily subcultures without antibiotic presence. To investigate the hypothesis that the acquisition of resistance causes a fitness cost to the bacterial cells and to estimate the therapeutic response of meropenem treatment, we performed experimental neutropenic thigh infections. Three meropenem dosing schemes were used, as there is a paucity of data on *A. baumannii *infections treated with meropenem, and the only scheme previously applied on *A. baumannii* (20 mg/L, reference [11]) exhibited very poor therapeutic responses in our preliminary experiments. 

The results of the experimental infections suggested that heterogeneously grown *A. baumannii* subpopulations have lower virulence compared with the clinical isolates, as 9/15 untreated animals infected by persisters or heteroresistant subpopulations survived, while all untreated mice infected by clinical isolates died. These results probably reflect a fitness cost conferred by mutations related to the expression of the heterogeneous mode of growth against meropenem. The lower virulence of the phenotypic heterogeneous subpopulations is also reflected by the observation that the lowest meropenem dosage (20 mg/kg/8 h) conferred survival of 14/15 mice infected by persisters or heteroresistant subpopulations in contrast with only 7/15 mice infected by clinical isolates to survive. Finally, the infection outcome under meropenem treatment was overall not correlated with the meropenem MICs, even in stably resistant heterogeneous populations, further suggesting their impaired virulence. 

## 3. Experimental

### 3.1. Study Isolates and Susceptibility Testing

The study included five *A. baumannii* clinical isolates from our collection and the *E. coli* ATCC 25922 and *P. aeruginosa* ATCC 27853 strains as controls for the experimental infections and the phenotypic assays, respectively. Susceptibility status to β-lactams, aminoglycosides, quinolones and colistin was performed by the Vitek2 automated system (BioMerieux, Marcy l’ Etoile, France) and disk diffusion [18]. Agar dilution meropenem MIC testing [18] was performed for the clinical isolates and for subpopulations grown at the highest meropenem concentrations in population analysis assays. 

### 3.2. PFGE and PCR Assays

The genetic relationship of the isolates was tested by PFGE of *Apa*I-digested genomic DNA [7], and the banding patterns were compared visually using previously proposed criteria [19]. PCR for the intrinsic *bla*_OXA-51_-like allele and for genes encoding known class B and D carbapenemases were performed as described previously [20,21,22]. 

### 3.3. Population Analyses

Population analyses using meropenem were performed in triplicate for all isolates. Phenotypically heterogeneous subpopulations were yielded by spreading approximately 10^8^ bacterial CFU on Mueller-Hinton agar plates with meropenem concentrations ranging from 0.5 to 32 mg/liter and incubating the plates for 48 h. Colonies grown in the highest drug concentration (heterogeneous subpopulations) were tested for meropenem MIC directly from the population analysis plates and after one week of daily subcultures in antibiotic-free medium to test for the stability of the phenotype. One colony of the seventh subculture for each strain (Ab1h, Ab2h, Ab3h, Ab4h and Ab5h) was selected for use in the thigh-infection model, along with the respective clinical isolates (Ab1, Ab2, Ab3, Ab4 and Ab5).

### 3.4. Growth Curves

Growth curves were determined by diluting 0.1 mL of overnight Mueller-Hinton broth culture of the clinical isolates and heterogeneously resistant subpopulations in 15 mL of broth followed by incubation at 37 °C under constant shaking. The optical density of a 1 mL aliquot of each broth culture was determined at each hour for 16 h.

### 3.5. Thigh Infection Model

Animal studies were approved by the Ethics Committee of Medical School, University of Thessaly, and conformed to the European Union guidelines. In each model, six-week-old, specific-pathogen-free, female Bagg inbred albino c-strain (BALB/c) mice (12-poster) weighing 23 to 27 g were used in each test group [23]. Mice were rendered neutropenic (neutrophils < 100/μL) by intraperitoneal cyclophosphamide on day 4 (150 mg/kg) and on day 1 (100 mg/kg) before thigh infection [24] and were anaesthetized with ketamine/xylazine, and thigh infections were produced by injecting 0.1 mL of bacterial suspension of 10^7^ CFU/mL for each study and control isolate, 2 h before giving antibiotic therapy, as described previously [25]. The infections were done in triplicate for all isolates. After mice were infected, they were given meropenem treatment for 24 h. As therapeutic protocols of experimental infections caused by *A. baumannii* have been scarcely reported, we used three meropenem dosing schemes: (i) 20 mg/kg/8h that was applied previously in a study with *A. baumannii* isolates [11], (ii) 100 mg/kg/12 h [12] and (iii) 400 mg/kg/8 h [13], which were both applied on mice infections caused by *P. aeruginosa*. Equal numbers of animals remained untreated as controls. Mice either died from infection or were sacrificed at 24 h; thigh muscles were homogenized in 10 mL of saline, serially diluted and cultured quantitatively after serial dilutions, for CFU determination. The level of detection of this assay was 100 CFU/thigh. When no organisms were cultured from the thighs, the number of CFU was arbitrarily set at 100 for further calculations. The thigh CFU count was expressed as log10 CFU/thigh muscle. A *t*-test was used for statistical analysis. For all experiments, a *p*-value of ≤0.05 was considered indicative of statistical significance. All statistical analyses were performed using Minitab software (version 13.31). 

## 4. Conclusions

In conclusion, *in vitro* meropenem exposure of *A. baumannii* isolates may induce phenotypic expression of heterogeneous resistance. This phenomenon is reversible for some isolates (persisters), but could also be permanent when associated with stable changes in the genome of *A. baumannii* (heteroresistance) [9]. However, the clinical importance of this phenomenon remains unknown. Our results indicate that the effect of the meropenem heterogeneous growth to the infectious process seems to be equivocal, as the virulence of the heterogeneous isolates is lower than that of the clinical isolates, while the meropenem treatment outcome seems not to be affected due to the elevated meropenem MICs.
